# Yellow fever in Ghana: Predicting emergence and ecology from historical outbreaks

**DOI:** 10.1371/journal.pgph.0003337

**Published:** 2024-10-21

**Authors:** Seth D. Judson, Ernest Kenu, Trevon Fuller, Franklin Asiedu-Bekoe, Alberta Biritwum-Nyarko, Lee F. Schroeder, David W. Dowdy

**Affiliations:** 1 Department of Medicine, Johns Hopkins University School of Medicine, Baltimore, Maryland, United States of America; 2 Department of Epidemiology, University of Ghana School of Public Health, Accra, Ghana; 3 Institute of the Environment and Sustainability, University of California, Los Angeles, California, United States of America; 4 Disease Surveillance Department, Ghana Health Service, Accra, Ghana; 5 Policy, Planning, Monitoring & Evaluation Division, Ghana Health Service, Accra, Ghana; 6 Department of Pathology, University of Michigan, Ann Arbor, Michigan, United States of America; 7 Department of Epidemiology, Johns Hopkins Bloomberg School of Public Health, Baltimore, Maryland, United States of America; New York University Grossman School of Medicine, UNITED STATES OF AMERICA

## Abstract

Understanding the epidemiology and ecology of yellow fever in endemic regions is critical for preventing future outbreaks. Ghana is a high-risk country for yellow fever. In this study we estimate the disease burden, ecological cycles, and areas at risk for yellow fever in Ghana based on historical outbreaks. We identify 2387 cases and 888 deaths (case fatality rate 37.7%) from yellow fever reported in Ghana from 1910 to 2022. During the approximately 30-year periods before and after implementation of routine childhood vaccination in 1992, the reported mean annual number of cases decreased by 80%. The geographic distribution of yellow fever cases has also changed over the past century. While there have been multiple large historical outbreaks of yellow fever in regions throughout Ghana, recent outbreaks have originated in northern regions. Comparing the locations where yellow fever outbreaks have emerged, we find patterns with seasons and different ecological transmission cycles. Using an ecological niche modeling framework, we predict areas in Ghana that are similar to where prior yellow fever outbreaks have originated based on temperature, precipitation, vegetation, and human population density. We find that these predictions differ depending on the ecological cycles of outbreaks. Ultimately, these findings and methods could be used to inform further subnational risk assessments for yellow fever in Ghana and other high-risk countries.

## Introduction

**Fig 1:**
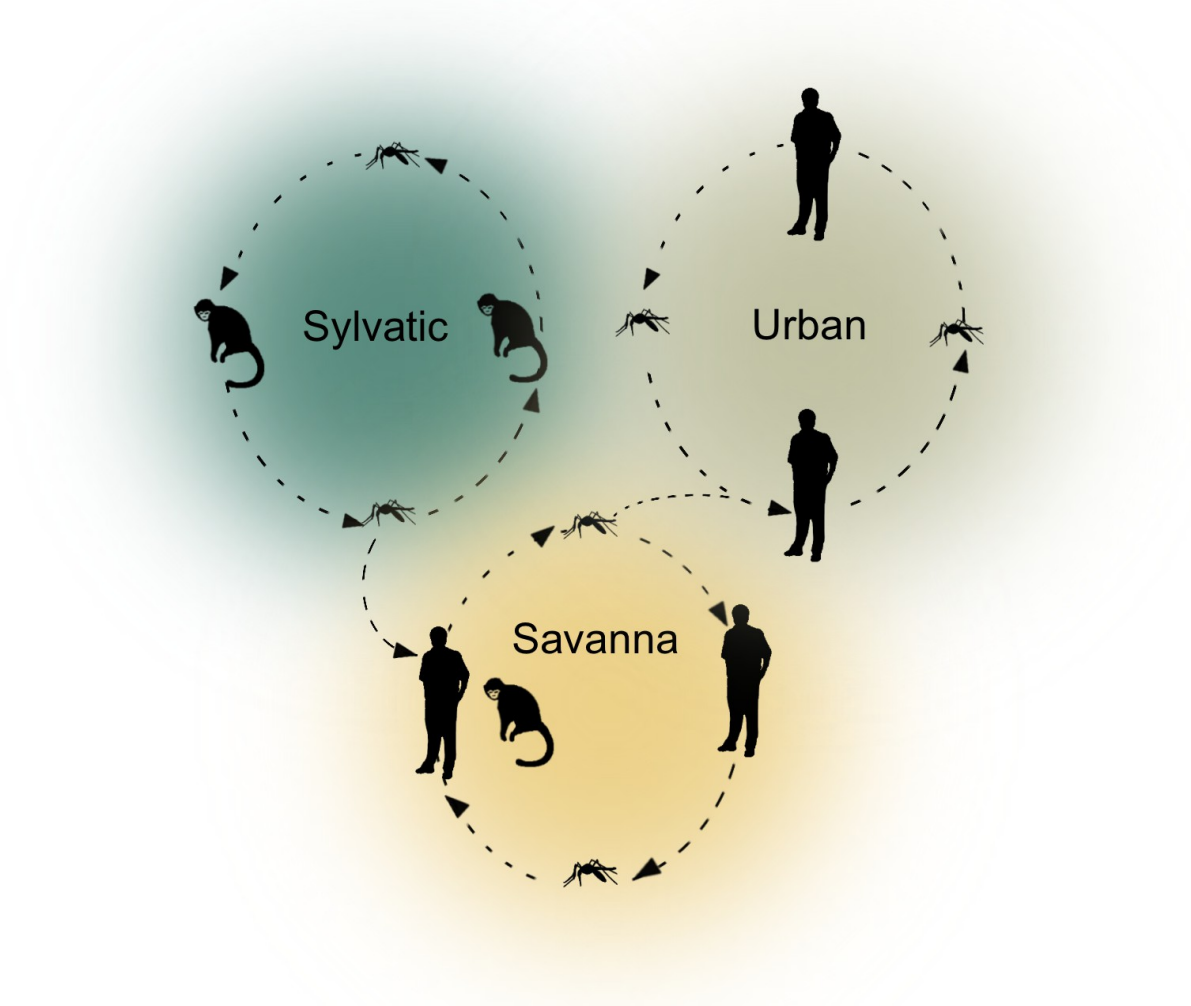
Ecological transmission cycles of yellow fever in Africa. The three ecological transmission cycles for yellow fever in Africa and Ghana are shown. The sylvatic cycle involves circulation of yellow fever virus between NHPs (predominantly Old World monkeys) with occasional transmission to humans. Vectors for this cycle in Ghana are arboreal *Aedes* mosquitoes such as *A*. *africanus* and *A*. *luteocephalus*. In the savanna cycle there are infections of humans and NHPs in rural and peridomestic settings via *A*. *aegypti* and other *Aedes* spp. such as *A*. *furcifer*. In the urban cycle there are larger outbreaks due to human-to-human transmission via *A*. *aegypti*.

Yellow fever (YF) outbreaks continue to occur in Africa and South America despite an effective vaccine having been created nearly a century ago due to gaps in immunization coverage and resource constraints [1]. Yellow fever is a viral hemorrhagic fever caused by yellow fever virus (YFV), a mosquito-borne flavivirus. In Africa, YFV circulates through three transmission cycles (Fig 1). In the sylvatic cycle, forest-dwelling *Aedes* spp. mosquitoes infect non-human primates (NHPs) and intermittently transmit YFV to humans after feeding on infected NHPs [2,3]. In the savanna or intermediate cycle, humans are infected by mosquitoes in forest border areas, and there is human-to-human and NHP-to-human transmission via mosquito vectors [2,3]. In the urban cycle, there is only transmission among humans from the anthropophilic mosquito *Aedes aegypti*, which breeds in water-containing vessels in urban sites [2,3]. While all three transmission cycles cause human cases of YF in Africa, it remains unknown how each cycle contributes to the burden of YF, limiting targeted interventions and predictive models [4,5].

Among infectious diseases in the WHO Africa Region (AFRO), YF caused the fifth most outbreaks in 2020 [6]. And while there are more cases of YF in West Africa compared to anywhere else in the world, a recent systematic review found only 12 studies of YF incidence and mortality within African countries [7]. Compounding the limited knowledge of the historical burden of YF, there are gaps in predicting future emergence of YF within African countries, and it is unknown how each of the three transmission cycles (sylvatic, savanna, and urban) contribute to YF outbreaks.

There is a contrasting situation in South America, where all outbreaks of YF in Brazil have been attributed to the sylvatic cycle since 1942 and there are known seasonal and climatic drivers of outbreaks, for example increased cases during agricultural seasons [8]. In addition, some species of New World NHPs develop lethal infections due to YFV, and the finding of moribund or dead NHPs can indicate an epizootic outbreak, signaling impending sylvatic YF outbreaks among humans [2]. In contrast, most YFV infections among Old World NHPs in Africa are believed to be inapparent or subclinical, and therefore outbreaks from sylvatic and savanna transmission occur without alerting indicators in Africa. Initial sylvatic or savanna outbreaks could lead to epidemics if the urban cycle subsequently becomes established, since the urban cycle causes sustained human-to-human transmission. Therefore, understanding the historical epidemiology and ecology of YF is crucial to predicting future emergence among YF-endemic African nations.

There is also concern for YF outbreaks within African countries to cause epidemics. In 2016 a YF outbreak in Angola spread to neighboring countries, and the epidemic response required more than 28 million YF vaccines, exhausting the global YF vaccine supply [9]. In response to this YF epidemic, the WHO, GAVI, and UNICEF developed the Eliminate Yellow Fever Epidemics (EYE) strategy with the aim of eliminating yellow fever epidemics by 2026 [9]. The core objectives of the EYE strategy are to (1) protect at-risk populations, (2) prevent international spread, and (3) contain outbreaks rapidly [10]. Central to achieving these objectives is understanding the regions and populations at greatest risk for YF outbreaks. Ghana is among the 27 countries identified by EYE as being high-risk for YF and has had multiple YF outbreaks.

Despite the introduction of routine YF vaccination for children in Ghana through the Expanded Program on Immunization (EPI) in 1992 and multiple preventative mass vaccination campaigns (PMVCs) since 2005, Ghana continues to experience YF outbreaks [11]. The most recent YF PMVC in November 2020 targeted 5.6 million individuals aged 10–60 across 81 districts in 14 regions [12]. However, a YF outbreak occurred from October 2021 to February 2022 with 70 confirmed cases and 35 deaths [13]. The origin and epicenter of the outbreak was in the West Gonja district of the Savannah region, starting among primarily unvaccinated nomadic populations who moved near a forest reserve, from which the outbreak spread to other regions [14,15]. These populations, residing in remote forested areas with limited healthcare access and frequent border crossings, pose challenges for vaccination efforts [11]. Additionally, the 2020 PMVC missed approximately 10% of the target population in rural, remote areas [12].

As of 2023, the WHO/UNICEF estimate that 88% of the population in Ghana has been vaccinated against YF [16]. While the YF vaccine is known to be highly effective, there is uncertainty about the duration of immunity [17]. Understanding the contexts of historical YF outbreaks in Ghana and identifying areas at risk for YF could therefore inform resource allocation for YF surveillance and vaccination campaigns. Modeling tools could aid risk assessment for YF by predicting areas at risk based on prior outbreaks. For zoonotic and vector-borne diseases like YF, the locations of cases, vectors, or hosts can be used to create spatial models which can be translated into risk maps [18]. For example, researchers have predicted the local risk for sylvatic YF in Brazil from ecological niche models (ENMs) based on human and NHP cases as well as ecological factors [19,20]. While there have been global ENMs for YF [21], a 2023 systematic review found no subnational models for YF in Africa [22].

Through combining epidemiological data from historical YF outbreaks with local ecological knowledge, we aim to develop an understanding of the ecological contexts in which YF occurs in Ghana. We compare the locations and conditions where YF outbreaks have emerged and conduct an exploratory ENM analysis, using confirmed human YF cases as inputs and multiple abiotic and biotic covariates as explanatory variables (S1 Appendix). Ultimately, our overall goals are to (1) review the historical epidemiology of YF outbreaks in Ghana, (2) assess contributions of different YF ecological cycles to these outbreaks, and (3) identify areas at risk for YF emergence based on this historical and ecological understanding.

## Methods

### Setting

The Republic of Ghana is a country in West Africa on the Gulf of Guinea, bordered by Côte d’Ivoire, Burkina Faso, and Togo. Ghana consisted of 10 regions until December 2018 when the Brong-Ahafo, Northern, Volta, and Western regions were split to make a total of 16 regions. For the purposes of this study, we used the current geographic boundaries of the 16 regions and 260 districts in Ghana. For outbreaks prior to 2019 with available geographic information, we re-classified these locations to align with the current terminology for regions and districts. We also compared outbreak locations by the five recently proposed agro-climatic zones for Ghana, which are named from north to south: Sudan Savannah, Guinea Savannah, Transition Zone, Forest, and Coastal [23]. These zones encompass geographic regions with similar temperature and precipitation patterns [23]. In general, the northern zones have uni-modal rainfall seasonality while the southern zones have bi-modal rainfall seasonality [23].

### Identifying YF outbreaks in Ghana

To identify YF outbreaks in Ghana and the locations of corresponding cases, we searched PubMed, EBSCO, Google Scholar, and Scopus for literature using the search terms “yellow fever” AND “Ghana.” We also searched the ProMed archives using the same search terms. To find WHO YF reports, we used the WHO’s Institutional Repository for Information Sharing (IRIS) and queried the Medical Subject Heading (MeSH) “yellow fever”, which included the Disease Outbreak News (DON) and Weekly Epidemiology Record (WER). This also included a 2000 WHO report which included YF case reporting among countries from 1950–1998 [24]. We also reviewed additional WHO documents, including a 2005 report on YF in Ghana [25] which was referenced in a review of arboviruses in Ghana [26]. Finally, we reviewed publicly available online reports from the Ghana Health Service (GHS) and the Ghana Weekly Epidemiological Report. The literature search was conducted up to October 20^th^ 2023. We collated all of the data from the aforementioned sources into a dataset of reported annual YF cases, deaths, and case fatality rates (CFR) for individuals in Ghana, as well as a dataset of YF outbreaks in Ghana by district including locations, month of outbreak onset, and reactive vaccination campaigns [27].

### Estimating annual YF cases and outbreak characteristics

For the period from 1910–1950, we extracted YF cases, deaths, and outbreak characteristics primarily from a single source [28]. For 1950–2022, we compared YF cases and deaths between multiple sources, including the primary literature and WHO reports. If there was a discrepancy in annual cases/deaths, we used the total cases/deaths reported in the primary literature, given delays between reporting cases and confirmatory testing.

We excluded cases from 2003 which were reported only in the WHO 2005 report [25] because we could not find these cases referenced in another source. We were also unable to find deaths reported for 31 cases in 2012, so these cases were excluded from our calculations of CFR [29]. If the source distinguished between reported and confirmed cases, as well as diagnostics used, we also included this information in our dataset. For our calculations of disease burden and CFR, we used overall reported cases and deaths.

### Categorizing YF outbreaks according to likely transmission cycles and seasons

For outbreaks with sufficient epidemiological information (including location details, diagnostic method, and vector surveillance), we categorized the outbreaks as likely occurring due to the urban, sylvatic, or savanna cycles [27]. If vector surveillance identified sufficient *A*. *aegypti* based on the indices described below, or if the outbreak was concentrated around urban dwellings or structures (i.e. wells or water storage containers) then these outbreaks were attributed to the urban cycle. Outbreaks where vector surveillance revealed insufficient *A*. *aegypti* and/or cases originated in forest or savanna habitat were attributed to the sylvatic or savanna cycles, respectively. When cases occurred in forest border areas and there was insufficient data to distinguish between the cycles or possible overlap, we refer to these as sylvatic/savanna. If there were initial cases in rural areas in forest/savanna habitats that were followed by many cases in urban areas, we classified these outbreaks as sylvatic/savanna to urban.

For categorizing urban YF, we considered the following vector indices: (1) house index (% of houses with at least one positive breeding place), (2) container index (% containers with *A*. *aegypti* larvae), and (3) Breteau index (# of positive larval breeding places per 100 houses) [30,31]. There is a high risk for urban YF when the house index > 35, container index > 20, and Breteau index > 50 [30,31]. If the Breteau index is between 5 and 50, then there is considered to be sufficient *A*. *aegypti* to cause an outbreak. There is unlikely to be urban transmission of YFV when the house index <4, container index <3, and Breteau index <5 [30,31].

We also categorized the season during the month when outbreaks were reported to have started. Seasons were categorized as either rainy or dry depending on (1) descriptions in the primary literature and (2) month of onset and corresponding rainfall pattern based on the agro-climatic zone [23].

### Georeferencing locations of YF emergence

In order to compare the locations and conditions associated with the emergence of YF outbreaks in Ghana, we first identified the specific locations and districts from the beginning of outbreaks. We refer to these specific locations, which represent the coordinates where one or more laboratory confirmed cases of YF occurred at the beginning of an outbreak, as “occurrences”. Our methods for obtaining, georeferencing, and processing data for these occurrences are fully described in the S1 Appendix. For our modeling analyses, we included occurrences that occurred since 1955. This timeframe was chosen because YF immunization became required for foreigners visiting Ghana in 1945, likely changing outbreak dynamics, and a panel of YF experts determined that one of the most important risk factors for YF in the African region was confirmed YF cases since 1960 [3]. We categorized the transmission cycles of occurrences as described above. For our analyses we excluded occurrences that were solely due to the sylvatic cycle since these could only be georeferenced to town of residence and infection likely occurred further away in forest areas.

Through this process we identified 23 occurrences for YF in Ghana from 1955–2022 (Table 1). We categorized 9 of these occurrences as being due to the urban cycle and 14 from the savanna cycle. Additional details on the occurrences are available in the online dataset and S1 Appendix. To compare the regional distribution of YF cases as well as districts where YF outbreaks have originated, we created maps in ArcGIS Pro (https://www.esri.com).

**Table 1:** Select YF outbreaks and cases in Ghana with epidemiologic details from 1910–2022.

Year	Region	Initial location	Agro-climatic zone	Suspected Cycle	Details	Ref
1910	Western	Sekondi	Coastal	urban	cases in confined area near commercial waterfront/dwellings	[28]
1912	Greater Accra	Accra	Coastal	urban	cases localized to an urban area around a mission	[28]
1926	Eastern	Nsawam	Forest	urban	outbreak confined within town; “aedes index” 89%	[28]
1927	Greater Accra	Accra	Coastal	urban	outbreak around particular dwellings	[28]
1927	Eastern	Larteh	Forest	urban	“aedes index was high”	[28]
1937	Greater Accra	Shai, Krobo, Accra	Coastal	urban	localized outbreak around a well with *A*. *aegypti*	[28]
1955	Bono East	Kintampo*, Kadelso*, Kunso*	Transition	sylvatic/savanna	no *A*. *aegypti* found, *A*. *africanus* found to be widespread	[32]
1969	Northern	Pong-Tamale*	Guinea Savannah	savanna	only 2/246 houses with *A*. *aegypti* larvae	[33]
1969	Upper East	Bolgatanga*	Sudan Savannah	urban	house index 11%	[33]
1970	Eastern	Akim-Manso*, Asikasu*, Akwatia*	Forest	urban	*A*. *aegypti* found breeding in all towns, house index as high as 50%	[33]
1977	Upper West	Jirapa*	Sudan Savannah	urban	house index 9.1%, container index 5.9%, Breteau 14%	[30]
1978	Eastern	Maase*	Forest	urban	house index 36.4%, container index 38%, Breteau index 96%	[30]
1978	Volta	Hohoe*, Kpandu*	Forest/Guinea Savannah	urban	house index 4–16%, container index 3–7%, Breteau index 4–16%	[30]
1983	Savannah	Damongo*	Guinea Savannah	sylvatic/savanna to urban	laboratory confirmed deaths of baboons at Mole reserve, subsequent urban spread	[34]
2006	Bono East	Kountaya village	Guinea Savannah	sylvatic	single case in village near forest	[35]
1996	Upper East	Bawku*	Sudan Savannah	urban	many cases in urban area, underwent vector control	[36]
2011	Upper East	Kassena-Nankana West	Sudan Savannah	sylvatic	index case went to a farm in a forest border	[37]
2021	Savannah	West Gonja*	Guinea Savannah	savanna to urban	initial cases in savanna habitat then urban spread	[14]

*Occurrences used in modeling analyses.

Additional categorized outbreaks and cases in online dataset [27].

### Comparing ecological factors

We compared factors associated with YF emergence based on the occurrence locations above and multiple covariates (Table 2). These covariates were identified as factors that could influence the ecological cycles of YF in Ghana. Previous models of YF have shown that temperature, precipitation, vegetation/landcover, elevation, human population density, and NHP richness are associated with YF in South America [20,38]. Increased NHP species richness and human population density were found to be associated increased risk of sylvatic YF in Brazil, with decreasing risk at the highest human population density levels [20]. Temperature, precipitation, and vegetation have been associated with seasonal cases of YF in Africa, given the influences these variables have on transmission via mosquitoes [39]. Therefore, we considered these variables as potential covariates for our analyses. This included (1) abiotic covariates: 19 bioclimatic variables and elevation (WorldClim 2.1, https://www.worldclim.org) [40] and (2) proxies for biotic covariates: Normalized Difference Vegetation Index (NDVI) (https://land.copernicus.eu/en/products/vegetation/normalised-difference-vegetation-index-v3-0-1km) [41], NHP species richness (https://sedac.ciesin.columbia.edu/data/set/species-global-mammal-richness-2015) [42], and (3) human population density (https://sedac.ciesin.columbia.edu/data/set/gpw-v4-population-density-adjusted-to-2015-unwpp-country-totals-rev11) [43].

**Table 2:** Explanatory covariates for YF habitat suitability models.

Covariate	Details	Resolution	Source
Annual mean temperature, annual precipitation, precipitation of the wettest quarter	Gridded climate data 1970–2000	30 arc seconds/1 km^2^	Worldclim 2.1
Elevation	Derived from the Shuttle Radar Tomography (SRTM) elevation data	30 arc seconds/1 km^2^	Worldclim 2.1
Species richness of Old World monkeys (Cercopithecidae)	Gridded values of the number of species of Cercopithecidae from the IUCN Red list	30 arc seconds/1 km^2^	Global Mammal Richness Grids, 2015 Release (2013) (SEDAC)
NDVI	Normalized Difference Vegetation Index 2020	30 arc seconds/1 km^2^	Copernicus NDVI Version 3 (2020)
Human population density	2020 UN adjusted population density	30 arc seconds/1 km^2^	UN WPP-Adjusted population density v4.11 (SEDAC)

To identify potential NHPs that could propagate the sylvatic/savanna cycles, we referenced the IUCN Red List (https://www.iucnredlist.org/) and identified 17 species of NHPs in Ghana. Of these species, six had epidemiological and laboratory evidence of YF infection at the species level (*Cercopithecus mona*, *Colobus vellerosus*, *Erythrocebus patas*, *Pan troglodytes*, *Papio anubis*, *Perodicticus potto*), and three had evidence at the genus level (*Cercopithecus lowei*, *Cercopithecus petaurista*, and *Cercopithecus roloway*) [3]. Eight of these nine species are members of the Cercopithecidae family. The exception was *P*. *troglodytes*, which is not a common species in Ghana and is unlikely to have a significant role in YF ecology [3]. Therefore, we evaluated species richness of Old World monkeys (Cercopithecidae) as a covariate.

Full details about the covariate selection process are available in S1 Appendix. We removed covariates that had a correlation coefficient of >|0.7| [44]. We also removed bioclimatic variables that were known to have discontinuities in sub-Saharan Africa [45]. The final covariates considered in the model selection process were population density, NDVI, elevation, NHP species richness, annual mean temperature (BIO1), annual precipitation (BIO12), and precipitation of the wettest quarter (BIO16).

### Modeling habitat suitability for YF emergence

To explore suitable habitats for YF emergence, we built on a previously described approach that is optimized for small sample sizes to identify habitat suitability using ENMs [46,47]. The chosen methodology and covariates were also similar to those used to model YF in Brazil [20], but modified for both a small sample size and to avoid over-fitting. We used Maximum Entropy Species Distribution Modeling (Maxent) [48], a machine learning algorithm commonly used to predict the distribution of a species based on presence locations as input and multiple ecological covariates. We chose Maxent given that it has outperformed other modeling algorithms for small sample sizes with presence-only data, including in environments in Africa [49]. We developed our Maxent models using R version 4.4.0 with the R package ENMEval v2.0.4 [50] which uses maxnet from the maxnet package v01.4; maxnet fits Maxent models using glmnet [51] (S1 Appendix). Our aim was to model the environments suitable for YF emergence in Ghana, as well as to explore the relative importance of different abiotic, biotic, and human covariates. Therefore, instead of making the stringent assumptions to interpret Maxent models as ecological niches, we instead built our models to explore factors related to habitat suitability for YF emergence [52].

We created one set of models with all 23 YF occurrences undifferentiated by ecological cycle, to predict areas with habitat suitability for YF irrespective of cycle. We hypothesized that factors influencing YF risk may differ by ecological transmission cycle. Given that the sylvatic and savanna cycles involve YFV co-circulation in rural forest habitats and forest-savanna ecotones with NHPs, whereas the urban cycle involves only circulation among humans in urban areas, we anticipated that the sylvatic and savanna cycles would be associated with a higher density of green vegetation (as measured by NDVI), increased NHP species richness, and lower human population density compared to the urban cycle. Therefore, we created another set of models which excluded the 9 urban occurrences to characterize the habitats where recent YF outbreaks have originated in the savanna cycle. Given sample size limitations we were unable to make reliable models exclusively for the sylvatic or urban cycles. The full steps for data collection, processing, model calibration and evaluation are available in the S1 Appendix.

## Results

**Fig 2:**
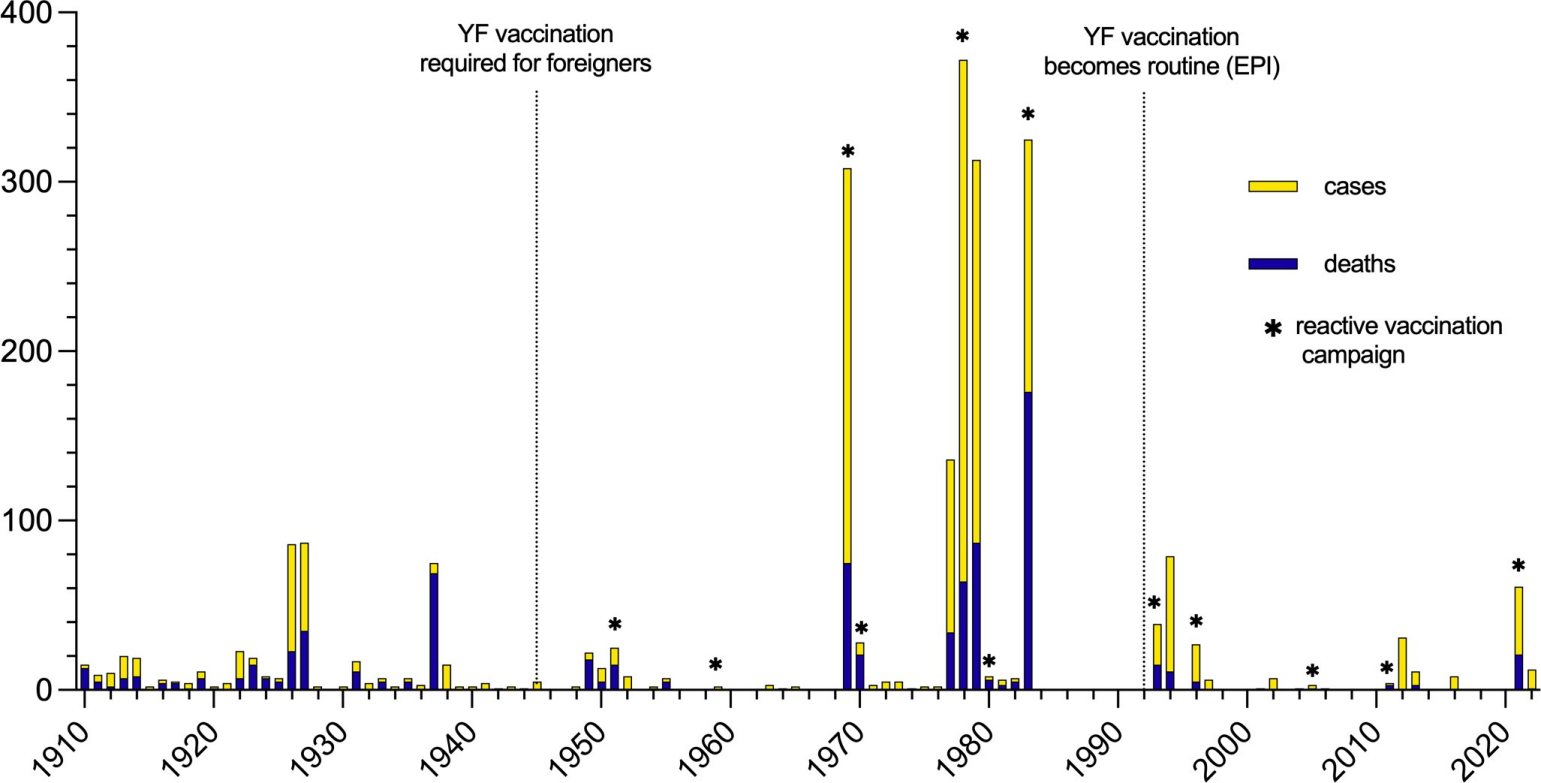
Annual yellow fever cases and deaths reported in Ghana 1910–2022. The annual yellow fever cases and deaths reported in Ghana are shown from the first confirmed outbreak in 1910 until the most recent outbreak in 2021–2022. Also shown are the years when reactive YF vaccination campaigns and immunization policies were initiated.

We identified 2387 cases and 888 deaths due to YF in Ghana from 1910 to 2022 (Fig 2). The overall mean CFR was 37.7% (excluding cases from 2012 since deaths were not reported). The first definitive outbreak of YF in Ghana occurred in 1910. From 1910–1960 there were 569 cases and 335 deaths (CFR 58.9%). In 1945 YF vaccination became required for foreigners in Ghana, and the first reactive mass vaccination campaign for YF in Ghana occurred in 1951. We identified 12 reactive vaccination campaigns from 1951–2022 (Fig 2).

**Fig 3:**
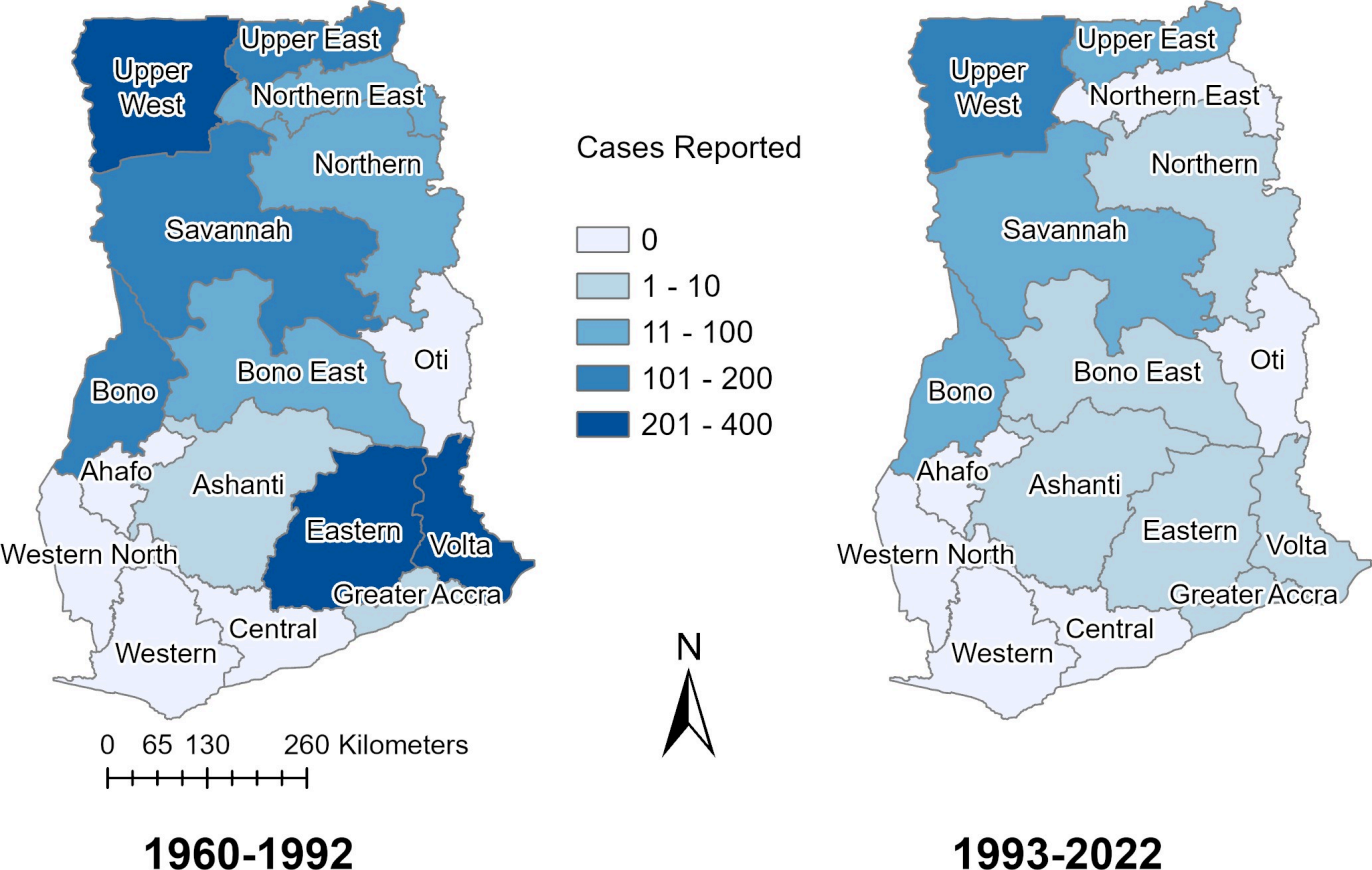
Yellow fever cases, reported by region, in Ghana before and after routine childhood YF vaccination. Reported YF cases that could be located on a regional level in Ghana are shown for the periods before and after routine childhood YF vaccination, (1960–1992 and 1993–2022, respectively). The regions of historical cases have been updated to match the current terminology for the 16 regions in Ghana. The region shapefile layers used to create the map of Ghana were obtained from the Humanitarian Data Exchange (https://data.humdata.org/dataset/cod-ab-gha?).

**Fig 4:**
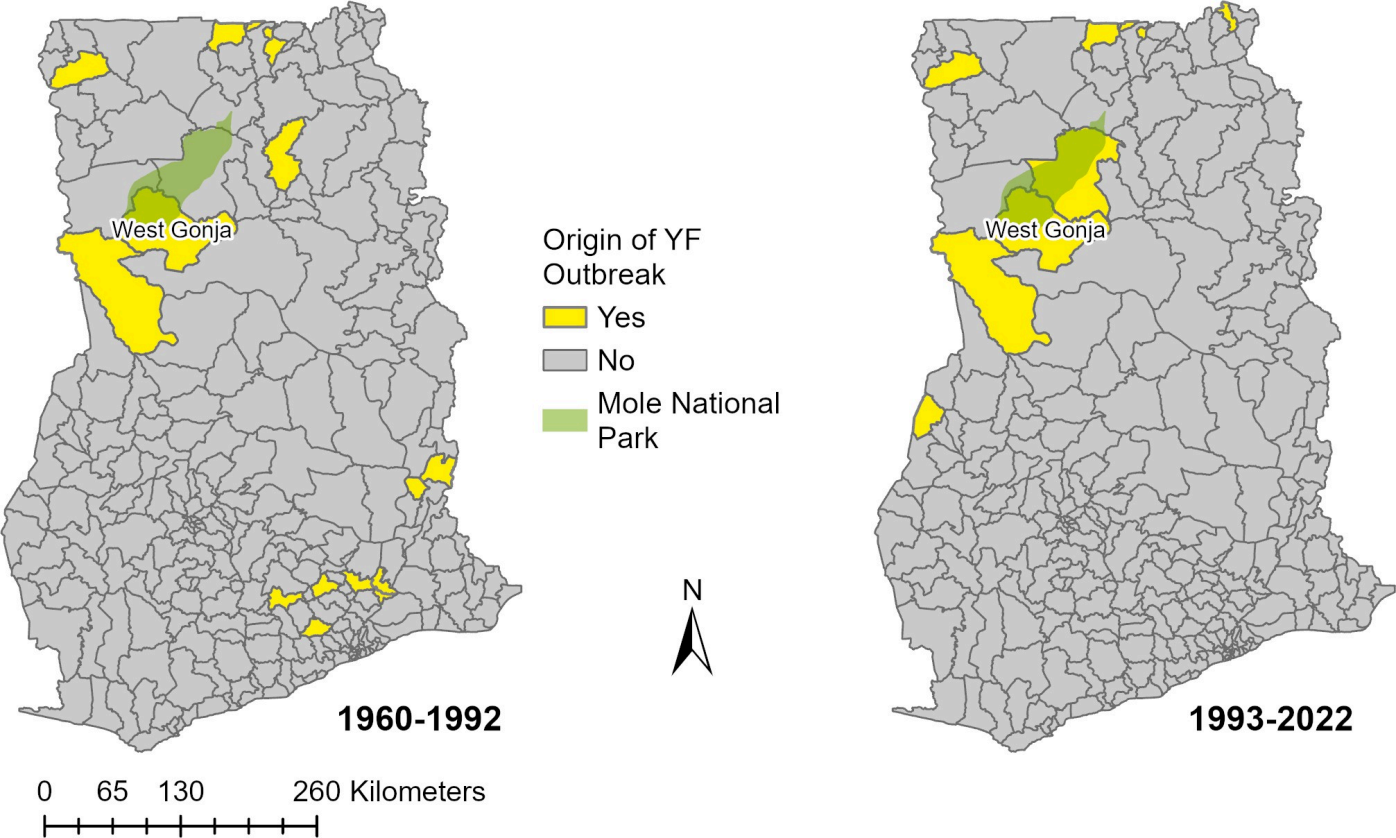
Yellow fever outbreak origins by district in Ghana. The districts where reported YF outbreaks were suspected to originate in Ghana are shown for the periods before and after routine childhood YF vaccination, (1960–1992 and 1993–2022, respectively). Also shown is Mole National Park and the West Gonja District where the 2021 and 1983 outbreaks were suspected to originate. The district shapefile layers used to create the map of Ghana were obtained from the Humanitarian Data Exchange (https://data.humdata.org/dataset/cod-ab-gha?). The extent of Mole National Park was extracted from a shapefile of Ghana forest reserves (https://data.gov.gh/dataset/shapefiles-forest-and-game-reserves-ghana-2010).

We successfully identified the regions in which 1739/1802 (96.5%) of reported cases from 1960 to 2022 occurred (Fig 3). From 1960–1992 there were 1527 cases and 492 deaths (CFR 32.2%) that were reported in 11 of Ghana’s 16 regions. The greatest number of YF cases were reported in Upper West (n = 384), Volta (n = 342), Eastern (n = 267), Upper East (n = 163), Savannah (n = 124), and Bono (n = 104) regions. Comparing the approximately 30-year periods pre and post implementation of routine childhood YF vaccination in 1992, the mean annual number of YF cases fell by 80%. From 1992–2022 there were 291 cases and 61 deaths (CFR 23.5%) with the highest number of cases in the Upper West (n = 135), Savannah (n = 45), and Upper East (n = 33) regions and few cases in the southern regions. The districts where YF outbreaks were suspected to originate in Ghana based on primary/index cases are shown in Fig 4.

We identified certain outbreaks and cases that could be categorized as likely due to urban, savanna, and sylvatic cycles in Ghana (Table 1). Of 13 outbreaks with available epidemiological and entomological data that occurred between 1910 and 1979, we estimated that 11 likely corresponded to the urban cycle. The 1983 outbreak and 2021–2022 outbreak seem likely to have originated in the sylvatic/savanna cycle and then spread through the urban cycle. In addition to these outbreaks there have been sporadic individual cases likely from the sylvatic/savanna cycles.

Among YF outbreaks in districts with temporal data, 11/11 outbreaks in northern regions in the Sudan and Guinea Savannah agro-climatic zones started during the dry season/end of rainy season. In contrast, urban outbreaks during 1970 and 1978 in the Eastern and Volta regions in the Forest agro-climatic zone were identified during the rainy season.

The predictions based on the models of habitat suitability for YF emergence are shown in S1 Fig. The optimal model containing both urban and savanna occurrences included NDVI, population density, and precipitation in the wettest quarter. The selected model containing only savanna occurrences had the same covariates but also included annual precipitation. Both models predicted high habitat suitability for YF in the northern regions (Upper West, Upper East, Northern East, Northern, Savannah, and Bono East), while the model with only savanna cases also predicted high habitat suitability in the Bono and Volta regions. Based on the response curves of the models (S2 Fig), the model containing only the savanna cycle predicted higher habitat suitability for YF in areas with relatively lower human population density, higher density of vegetation (NDVI), and different seasonal precipitation patterns compared to the model containing the urban cycle.

## Discussion

Our analysis estimates the historical epidemiology and ecology of YF in Ghana as well as areas at potential risk for future emergence of YF. We found that the disease burden and geographic distribution of YF in Ghana have changed substantially over the past century. While historical outbreaks occurred in coastal and southern cities, recent outbreaks have originated in rural northern regions. The requirement for YF immunization in foreigners starting in 1945 and the introduction of routine childhood YF vaccination in 1992 likely contributed to these changes.

The overall mean CFR we calculated for YF in Ghana (37.7%) was similar to the mean CFR from a recent meta-analysis of global YF cases (39%) and severe cases in African countries (36%) [7]. Given that there is limited surveillance and diagnostic capacity for YF in Ghana, our data likely underestimate the true burden of YF (while overestimating the true CFR) given that mild or subclinical infections may not be detected. The YF cases in our study more likely reflect severe YF, and it is estimated that ~15% of YF cases are from severe YF. If only severe YF cases were captured in our dataset, this would suggest that over 15,000 cases of YF have occurred in Ghana since 1910 –a number that may be even higher given historical and current limitations in diagnosing YF. Both the number of cases and the case-fatality of YF have declined in Ghana since 1992, likely in part due to introduction of routine childhood YF vaccination in 1992. Nevertheless, YF outbreaks continue to occur in Ghana, highlighting the need to understand which areas and populations are at greatest risk.

During the first half of the 20^th^ century, the most YF outbreaks in Ghana were detected along coastal regions and periodically in the north, with large outbreaks occurring roughly every 10 years [28]. Relatively higher population density and the presence of susceptible foreign populations likely contributed to these urban YF outbreaks. Detection bias also likely contributed to these observations given differences in accessibility to diagnostics and reporting. The requirement for YF vaccination among foreigners in 1945, local reactive vaccination campaigns, and increasing diagnostic recognition likely contributed to the changing distribution of YF outbreaks with greater recognition of outbreaks in more remote northern regions.

In the context of increasing vaccination coverage for YF in Ghana, recent outbreaks have originated among unvaccinated populations in rural areas and border districts in the Guinea Savannah and Sudan Savannah agro-climatic zones. These areas may have more nomadic pastoralist communities and immigrants from neighboring countries, potentially contributing to lower vaccination coverage. These populations live near environments where the appropriate vectors, NHPs, and human hosts are present to maintain the sylvatic or savanna cycles. Therefore recent YF outbreaks appear to have originated in the sylvatic and savanna cycles, which could transition into the urban cycle if there are sufficient susceptible hosts.

Environmental change is predicted to increase habitat suitability for *A*. *aegypti* due to increased temperatures, changing precipitation patterns, and urbanization, fueling the spread of sylvatic/savanna YF to urban outbreaks [53,54]. Habitat disruption could also lead to increased contact between humans, NHPs, and vectors for YF. We found that YF outbreaks in northern regions originated during the dry season. The Sudan Savannah and Guinea Savannah agro-climatic zones have longer dry seasons [23], leading to increased water storage that provides breeding sites for *A*. *aegypti* which could cause outbreaks via the urban and savanna cycles [30]. These arid conditions have spread north and south in Ghana in the setting of climate change [23], potentially creating more suitable habitat for *A*. *aegypti* and YF transmission. In contrast outbreaks in the southern Forest agro-climatic zone occurred during the rainy season which occurs more frequently in this zone and could cause water to pool, creating mosquito breeding sites [30]. Further understanding the effects of environmental change on local vectors and hosts for YF will therefore be important for assessing future outbreak risk.

We used confirmed human cases and ecological covariates to identify likely suitable habitats for the emergence of YF in Ghana. The models do not represent the distribution or ecological niche of YFV, but instead represent habitats with similar conditions to those where prior YF cases occurred. Given that YF can start in the sylvatic/savanna cycles and then spread through the urban cycle, we also created a set of models excluding confirmed urban YF cases to provide a prediction of habitats where outbreaks may originate. This model is a proxy for the habitats similar to those where savanna cases have occurred, which appears to be the likely origin of recent and future YF outbreaks in Ghana.

To our knowledge, this is the first time that the different ecological cycles of YF have been integrated into spatial models in Africa. Overall these models illustrate the important differences in habitats and locations where YF may occur in Ghana. For example, while precipitation, vegetation, and human population density are important covariates in both sets of models, relatively higher human population density and lower vegetation density were associated with models that included outbreaks from the urban cycle–reflecting that the urban cycle occurs in urban environments with a higher density of susceptible human hosts compared to the sylvatic and savanna cycles, which occur in rural forest areas and forest-savanna ecotones. Predictive models that do not distinguish between the ecological cycles of YF risk misidentifying areas at risk for emergence. For instance, our models excluding urban cases reflect habitat similar to where the recent YF outbreaks have recently originated in Ghana and could be a better predictor for where future outbreaks may originate. In contrast, the set of models including urban occurrences is more appropriate for predicting areas where YF may spread through the urban cycle. Therefore, it is important that researchers think carefully about the goals and inferences of their models based on YF transmission cycles. Additionally, further local data on YF outbreaks and surveillance are needed to build on this approach and apply it in other settings.

The Upper West, Savannah, and Upper East regions have experienced the most YF cases since routine immunization. Our models identified these regions as well as neighboring regions (North East, Northern, Bono, and Bono East) as being highly suitable for YF emergence. The West Gonja district where the 2021 YF outbreak originated is located near Mole National Park (Fig 4), which is the largest protected area in Ghana and is a likely suitable habitat for the NHPs and mosquitoes that could maintain the sylvatic and savanna cycles of YF. Mole National Park is also a tourist site where nomadic populations may work and also has surrounding pastoralist communities, and these groups may have lower vaccination coverage for YF [55]. The 1983 YF outbreak was also believed to have originated from this area given the finding of laboratory confirmed deaths of baboons with YF [34].

Reviewing the locations of historical YF emergence, we found additional locations with recurring cases. At least four outbreaks appear to have originated in the Jirapa district in the Upper West and three in Bawku Municipal district in the Upper East. During the 2021 outbreak, there were two cases from Tinga village in the Bole region, and given the distance from the epicenter of the outbreak it is possible that this could have been a separate YF emergence event. This village also had clinically diagnosed cases of YF in 1983. While there have been isolated cases of sylvatic/savanna YF since routine immunization, with index cases exposed to forest-savanna ecotones, these cases did not lead to sustained outbreaks. In contrast, a significant unvaccinated population likely contributed to the 2021–2022 outbreak. Therefore, identifying both susceptible populations as well as the habitat for YF emergence will be crucial for predicting areas at future risk.

There are multiple limitations to our analysis. We used reported YF cases from the literature and online reports, and there were relatively few laboratory confirmed historical cases. For our habitat suitability models, we used strict inclusion criteria of confirmed cases with georeferenced locations, limiting our analysis to 23 total occurrences. Given the small sample size there is risk for sampling biases. We attempted to reduce overfitting by using leave-one-out cross validation, simplified models, and a small number of covariates. Another limitation to this analysis is that it was done with presence only data and not presence-absence data. There was also sparse epidemiologic and entomologic surveillance data for most outbreaks. Vector indices were only available for some of the occurrences, so we had to make assumptions about certain outbreaks that lacked entomologic data. Similarly, it is possible that both urban and savanna cycles may occur together during outbreaks. For example, while the 2021 YF outbreak may have originated in the savanna cycle, it could have spread through dwellings via the urban cycle. We categorized this outbreak and similar outbreaks as originating from the savanna cycle since our goal was to model habitat for YF emergence. The alternative explanation, that this YF outbreak originated from the urban cycle of another outbreak, seemed less likely given lack of nearby preceding outbreaks. Overall, additional vector surveillance data are needed for further clarity. Additional data are also needed from other countries are needed to determine whether outbreaks originated in Ghana or spread from neighboring countries. Lastly, the covariates we chose for our models were intended to reflect habitat suitability via presence of the appropriate climatic conditions, environments, and hosts. To make models more reflective of relative risk for future outbreaks, they would need to include additional explanatory variables, most importantly local YF vaccination coverage.

Despite these limitations, our study has important implications for preparing for future outbreaks of YF in Ghana. For example, our analyses could be used to inform further risk assessment which could guide diagnostic testing, vector control, and vaccination campaigns. A committee of YF experts developed a WHO protocol for national risk assessment for YF [3]. However, the strategies in this protocol are resource intensive and involve sampling humans, vectors, and NHPs for YF. Our methods for estimating habitat suitability based on historical cases could be used to prioritize field surveillance for YF with the WHO risk assessment protocol [3]. Our findings also have important implications for models and risk maps for YF. While predictive models for YF risk have been made on a global scale [21,56,57], such models either contain sparse data from Ghana or do not predict subnational risk for YF. Additionally, these existing models do not distinguish between the different ecological cycles of YF in Africa, which may influence prediction and interpretation. Therefore, additional subnational data and local ecological knowledge are needed to create future risk maps for YF and other arboviruses if these predictions are to be informative for national policymakers [18].

While recent YF outbreaks and predicted habitat suitability are predominantly in the northern regions of Ghana, currently all YF diagnostic testing is done at the National Public Health Reference Laboratory in Korle Bu in the south of the country. Expanding YF diagnostic testing to the Zonal Public Health Laboratory in Tamale, which is close to recent YF outbreaks and predicted habitat suitability, could improve early detection of outbreaks. While the burden of YF has decreased since implementation of routine childhood vaccination in 1992, the 2021–2022 outbreak has also demonstrated the risk for future outbreaks among unvaccinated groups. Identifying unvaccinated nomadic populations in habitats that are suitable for YF emergence will be an important step for preventing future outbreaks. Overall, despite a century of research on YF, critical questions remain regarding the ecology, epidemiology, and emergence of YF in African countries. Addressing these research gaps and using this knowledge to inform public health interventions will be essential to improving health equity and preventing future YF epidemics.

## Supporting information

S1 Appendix (DOCX)

S1 Fig Predictions for YF models. The predictions based on the default cloglog output for the selected models containing (A) all 23 YF occurrences and (B) 14 occurrences from the savanna cycle are shown. Values closer to 1 indicate a higher relative suitability for YF based on the locations of confirmed human cases and covariates. The georeferenced confirmed YF occurrences are represented by points.
(PDF)

S2 Fig Response curves for YF models. The response curves above visualize the relationship between the probability of YF occurrence and covariates for each model. The shape of the curve indicates how relative suitability changes in response to the covariates. For example, the steeply downward sloping of the response curve for the savanna YF model with population density indicates a greater negative relationship between population density and savanna YF. Note that the standard units for each variable are used except for NDVI (which is rescaled from 0 to 250 by the data source rather than -1 to 1).
(PDF)

## References

[1] Lindsey NP, Horton J, Barrett ADT, Demanou M, Monath TP, Tomori O, et al. Yellow fever resurgence: An avoidable crisis? npj Vaccines 2022;7: 1–3. doi: 10.1038/s41541-021-00424-2 36323723 PMC9629880

[2] Monath TP, Vasconcelos PFC. Yellow fever. Journal of Clinical Virology. 2015;64: 160–173. doi: 10.1016/J.JCV.2014.08.030 25453327

[3] Risk assessment on yellow fever virus circulation in endemic countries. [cited 7 Nov 2023]. Available: https://www.who.int/publications-detail-redirect/WHO-HSE-PED-CED-2014-2.

[4] Gaythorpe KAM, Jean K, Cibrelus L, Garske T. Quantifying model evidence for yellow fever transmission routes in Africa. PLoS Comput Biol. 2019;15: e1007355. doi: 10.1371/journal.pcbi.1007355 31545790 PMC6779277

[5] Jean K, Hamlet A, Benzler J, Cibrelus L, Gaythorpe KAM, Sall A, et al. Eliminating yellow fever epidemics in Africa: Vaccine demand forecast and impact modelling. PLOS Neglected Tropical Diseases. 2020;14: e0008304. doi: 10.1371/journal.pntd.0008304 32379756 PMC7237041

[6] Regional Committee for Africa. Progress report on the implementation of the regional strategy for health security and emergencies 2016–2020: information document. World Health Organization. Regional Office for Africa; 2019. Report No.: AFR/RC69/INF.DOC/1. Available: https://apps.who.int/iris/handle/10665/331442.

[7] Nwaiwu AU, Musekiwa A, Tamuzi JL, Sambala EZ, Nyasulu PS. The incidence and mortality of yellow fever in Africa: a systematic review and meta-analysis. BMC Infectious Diseases. 2021;21: 1–11. doi: 10.1186/s12879-020-05706-z 34688249 PMC8536483

[8] Hamlet A, Ramos DG, Gaythorpe KAM, Romano APM, Garske T, Ferguson NM. Seasonality of agricultural exposure as an important predictor of seasonal yellow fever spillover in Brazil. Nature communications. 2021;12. doi: 10.1038/s41467-021-23926-y 34131128 PMC8206143

[9] World Health Organization. A global strategy to eliminate yellow fever epidemics (EYE) 2017–2026. Geneva: World Health Organization; 2018. Available: https://apps.who.int/iris/handle/10665/272408.

[10] Eliminate yellow fever epidemics (EYE) strategy 2017–2026. [cited 5 Mar 2023]. Available: https://www.who.int/initiatives/eye-strategy.

[11] WHO. Mid-term evaluation of the Global Strategy to Eliminate Yellow Fever Epidemics (‎EYE)‎ 2017–2026: Annexes. [cited 6 Aug 2024]. Available: https://www.who.int/publications/i/item/WHO-DGO-EVL-2023.6.

[12] Amponsa-Achiano K, Frimpong JA, Barradas D, Bandoh DA, Kenu E. Leveraging Lessons Learned from Yellow Fever and Polio Immunization Campaigns during COVID-19 Pandemic, Ghana, 2021—Volume 28, Supplement—November 2022—Emerging Infectious Diseases journal—CDC. Emerging infectious diseases. 2022;28: 232–237. doi: 10.3201/EID2813.221044 36502407 PMC9745221

[13] Bonney JHK, Sanders T, Pratt D, Agbodzi B, Laryea D, Agyeman NKF, et al. Molecular Characterization of Circulating Yellow Fever Viruses from Outbreak in Ghana, 2021–2022. Emerg Infect Dis. 2023;29(9):1818–1826. doi: 10.3201/eid2909.221671 37610174 PMC10461649

[14] World Health Organization. Disease Outbreak News; Yellow Fever—Ghana. 1 Dec 2021 [cited 1 Aug 2023]. Available: https://www.who.int/emergencies/disease-outbreak-news/item/yellow-fever—ghana.

[15] Word Health Organization. Yellow Fever—West and Central Africa. In: Disease Outbreak News [Internet]. 23 Dec 2021 [cited 17 Jan 2024]. Available: https://www.who.int/emergencies/disease-outbreak-news/item/yellow-fever—west-and-central-africa.

[16] WHO Immunization Data portal—Yellow Fever (YF) vaccination coverage. In: Immunization Data [Internet]. [cited 6 Aug 2024]. Available: https://immunizationdata.who.int/global/wiise-detail-page/yellow-fever-(yf)-vaccination-coverage?CODE=Global&YEAR=.

[17] Staples JE, Barrett ADT, Wilder-Smith A, Hombach J. Review of data and knowledge gaps regarding yellow fever vaccine-induced immunity and duration of protection. npj Vaccines 2020 5:1. 2020;5: 1–7. doi: 10.1038/s41541-020-0205-6 32655896 PMC7338446

[18] Judson SD, LeBreton M, Fuller T, Hoffman RM, Njabo K, Brewer TF, et al. Translating Predictions of Zoonotic Viruses for Policymakers. EcoHealth. 2017. doi: 10.1007/s10393-017-1304-3 29230614

[19] Aliaga-Samanez A, Real R, Segura M, Marfil-Daza C, Olivero J. Yellow fever surveillance suggests zoonotic and anthroponotic emergent potential. Communications Biology 2022 5:1. 2022;5: 1–12. doi: 10.1038/s42003-022-03492-9 35654842 PMC9163115

[20] de Thoisy B, Silva NIO, Sacchetto L, Trindade G de S, Drumond BP. Spatial epidemiology of yellow fever: Identification of determinants of the 2016–2018 epidemics and at-risk areas in Brazil. PLOS Neglected Tropical Diseases. 2020;14: e0008691. doi: 10.1371/journal.pntd.0008691 33001982 PMC7553304

[21] Shearer FM, Longbottom J, Browne AJ, Pigott DM, Brady OJ, Kraemer MUG, et al. Existing and potential infection risk zones of yellow fever worldwide: a modelling analysis. The Lancet Global Health. 2018;6: e270–e278. doi: 10.1016/S2214-109X(18)30024-X 29398634 PMC5809716

[22] Lim A-Y, Jafari Y, Caldwell JM, Clapham HE, Gaythorpe KAM, Hussain-Alkhateeb L, et al. A systematic review of the data, methods and environmental covariates used to map Aedes-borne arbovirus transmission risk. BMC Infectious Diseases. 2023;23: 708. doi: 10.1186/s12879-023-08717-8 37864153 PMC10588093

[23] Yamba EI, Aryee JNA, Quansah E, Davies P, Wemegah CS, Osei MA, et al. Revisiting the agro-climatic zones of Ghana: A re-classification in conformity with climate change and variability. PLOS Climate. 2023;2: e0000023. doi: 10.1371/journal.pclm.0000023

[24] WHO report on global surveillance of epidemic-prone infectious diseases. [cited 2 Dec 2023]. Available: https://www.who.int/publications-detail-redirect/WHO-CDS-CSR-ISR-2000.1.

[25] WHO. Yellow Fever Cases Reported in Ghana, 1950–2004. World Health Organization; 2005.

[26] Agboli E, Tomazatos A, Maiga-Ascofaré O, May J, Lühken R, Schmidt-Chanasit J, et al. Arbovirus Epidemiology: The Mystery of Unnoticed Epidemics in Ghana, West Africa. 2022. doi: 10.3390/microorganisms10101914 36296190 PMC9610185

[27] Judson S. Yellow Fever in Ghana: Predicting Emergence and Ecology from Historical Outbreaks (Datasets). figshare; 2024. doi: 10.6084/m9.figshare.24747165.v6 PMC11493279 39432459

[28] Scott D. Epidemic Disease in Ghana 1901–1960. London: Oxford Univesity Press; 1965.

[29] WHO. Yellow fever in Africa and South America, 2011–2012 = Fièvre jaune en Afrique et en Amérique du Sud, 2011–2012. Weekly Epidemiological Record = Relevé épidémiologique hebdomadaire. 2013;88: 285–296. 23909009

[30] Agadzi VK, Boatin BA, Appawu MA, Mingle JA, Addy PA. Yellow fever in Ghana, 1977–80. Bulletin of the World Health Organization. 1984;62: 577. 6333294 PMC2536325

[31] Joannides J, Dzodzomenyo M, Azerigyik F, Agbosu EE, Pratt D, Osei JHN, et al. Species composition and risk of transmission of some Aedes-borne arboviruses in some sites in Northern Ghana. PLOS ONE. 2021;16: e0234675. doi: 10.1371/journal.pone.0234675 34061882 PMC8168856

[32] Boorman JPT, Porterfield JS. A small outbreak of yellow fever in the Gold Coast. Transactions of The Royal Society of Tropical Medicine and Hygiene. 1957;51: 439–449. doi: 10.1016/0035-9203(57)90079-2 13468005

[33] Beausoleil EG, Mukhesee AB, Grant FC, Herron CA. Surveillance of yellow fever in Ghana, 1960 et 1970. ORSTOM Ser Entomol Med Parasitol. 1972;10: 99–101.

[34] P.A.K. ADDY KM and VKA. Recent Yellow Fever Epidemics in Ghana (1969–1983). EAST AFRICAN MEDICAL JOURNAL. 1986;63. 3769852

[35] World Health Organization. Weekly Epidemiological Record, 2008, vol. 83, 08 [full issue]. Weekly Epidemiological Record = Relevé épidémiologique hebdomadaire. 2008;83: 69–76.

[36] World Health Organization. Weekly Epidemiological Record, 1998, vol. 73, 46 [full issue]. Weekly Epidemiological Record = Relevé épidémiologique hebdomadaire. 1998;73: 353–360.

[37] WHO. Yellow fever in Ghana. In: Disease Outbreak News [Internet]. 3 Feb 2012 [cited 3 Dec 2023]. Available: https://www.who.int/emergencies/disease-outbreak-news/item/2012_02_03b-en.

[38] Servadio JL, Muñoz-Zanzi C, Convertino M. Environmental determinants predicting population vulnerability to high yellow fever incidence. R Soc Open Sci. 9: 220086. doi: 10.1098/rsos.220086 35316947 PMC8889195

[39] Hamlet A, Jean K, Perea W, Yactayo S, Biey J, Van Kerkhove M, et al. The seasonal influence of climate and environment on yellow fever transmission across Africa. PLOS Neglected Tropical Diseases. 2018;12: e0006284. doi: 10.1371/journal.pntd.0006284 29543798 PMC5854243

[40] Hijmans RJ, Cameron SE, Parra JL, Jones PG, Jarvis A. Very high resolution interpolated climate surfaces for global land areas. International Journal of Climatology. 2005;25: 1965–1978. doi: 10.1002/joc.1276

[41] Normalised Difference Vegetation Index 1999–2020 (raster 1 km), global, 10-daily–version 3. [cited 27 Apr 2024]. Available: https://land.copernicus.eu/en/products/vegetation/normalised-difference-vegetation-index-v3-0-1km.

[42] International Union for Conservation of Nature—IUCN, Center for International Earth Science Information Network—CIESIN—Columbia University. Gridded Species Distribution: Global Mammal Richness Grids, 2015 Release. Palisades, New York: NASA Socioeconomic Data and Applications Center (SEDAC); 2015. Available: doi: 10.7927/H4N014G5

[43] Center for International Earth Science Information Network—CIESIN—Columbia University. Gridded Population of the World, Version 4 (GPWv4): Population Density Adjusted to Match 2015 Revision UN WPP Country Totals, Revision 11. Palisades, New York: NASA Socioeconomic Data and Applications Center (SEDAC); 2018. Available: doi: 10.7927/H4F47M65

[44] Feng X, Park DS, Walker C, Peterson AT, Merow C, Papeş M. A checklist for maximizing reproducibility of ecological niche models. Nature Ecology & Evolution 2019 3:10. 2019;3: 1382–1395. doi: 10.1038/s41559-019-0972-5 31548646

[45] Booth TH. Checking bioclimatic variables that combine temperature and precipitation data before their use in species distribution models. Austral Ecology. 2022;47: 1506–1514. doi: 10.1111/aec.13234

[46] Pearson RG, Raxworthy CJ, Nakamura M, Townsend Peterson a. Predicting species distributions from small numbers of occurrence records: A test case using cryptic geckos in Madagascar. Journal of Biogeography. 2007;34: 102–117. doi: 10.1111/j.1365-2699.2006.01594.x

[47] Shcheglovitova M, Anderson RP. Estimating optimal complexity for ecological niche models: A jackknife approach for species with small sample sizes. Ecological Modelling. 2013;269: 9–17. doi: 10.1016/j.ecolmodel.2013.08.011

[48] Phillips SJ, Anderson RP, Schapire RE. Maximum entropy modeling of species geographic distributions. Ecological Modelling. 2006;190: 231–259. doi: 10.1016/j.ecolmodel.2005.03.026

[49] van Proosdij ASJ, Sosef MSM, Wieringa JJ, Raes N. Minimum required number of specimen records to develop accurate species distribution models. Ecography. 2016;39: 542–552. doi: 10.1111/ecog.01509

[50] Kass JM, Muscarella R, Galante PJ, Bohl CL, Pinilla-Buitrago GE, Boria RA, et al. ENMeval 2.0: Redesigned for customizable and reproducible modeling of species’ niches and distributions. Methods in Ecology and Evolution. 2021;12: 1602–1608. doi: 10.1111/2041-210X.13628

[51] Phillips SJ, Anderson RP, Dudík M, Schapire RE, Blair ME. Opening the black box: an open-source release of Maxent. Ecography. 2017;40: 887–893. doi: 10.1111/ecog.03049

[52] Merow C, Smith MJ, Silander JA Jr. A practical guide to MaxEnt for modeling species’ distributions: what it does, and why inputs and settings matter. Ecography. 2013;36: 1058–1069. doi: 10.1111/j.1600-0587.2013.07872.x

[53] Gaythorpe KAM, Hamlet A, Cibrelus L, Garske T, Ferguson NM. The effect of climate change on yellow fever disease burden in Africa. eLife. 2020;9: 1–27. doi: 10.7554/eLife.55619 32718436 PMC7386919

[54] Girard M, Nelson CB, Picot V, Gubler DJ. Arboviruses: A global public health threat. Vaccine. 2020;38: 3989–3994. doi: 10.1016/j.vaccine.2020.04.011 32336601 PMC7180381

[55] Inusah A-W, Collins G, Dzomeku P, Head Id M, Ziblim S-D. Knowledge, attitudes and practice towards yellow fever among nomadic populations: A cross-sectional study in yellow fever outbreak communities in Ghana. PLOS Global Public Health. 2023;3: e0000733. doi: 10.1371/journal.pgph.0000733 36962969 PMC10019665

[56] Jentes ES, Poumerol G, Gershman MD, Hill DR, Lemarchand J, Lewis RF, et al. The revised global yellow fever risk map and recommendations for vaccination, 2010: consensus of the Informal WHO Working Group on Geographic Risk for Yellow Fever. The Lancet Infectious Diseases. 2011;11: 622–632. doi: 10.1016/S1473-3099(11)70147-5 21798462

[57] Gaythorpe KAM, Hamlet A, Jean K, Ramos DG, Cibrelus L, Garske T, et al. The global burden of yellow fever. eLife. 2021;10. doi: 10.7554/ELIFE.64670 33722340 PMC7963473

