# Supplementary material for: Yellow fever in Ghana: Predicting emergence and ecology from historical outbreaks
Source: PLOS Glob Public Health. 2024 Oct 21;4(10):e0003337. doi: 10.1371/journal.pgph.0003337 (PMC11493279; doi:10.1371/journal.pgph.0003337)
Supplement: S1 Appendix — (DOCX) [file pgph.0003337.s001.docx]

**Supplemental Appendix**

**Ecological Niche Modeling**

*Georeferencing YF Occurrence data*

To obtain occurrences for our model input, we searched for laboratory confirmed cases of YF in Ghana from 1910 to 2022 and georeferenced these cases to town of residence or healthcare facility. We georeferenced locations using Fallingrain Global Gazeteer version 2.3 (http://www.fallingrain.com/world/GH/) and Google Earth (https://earth.google.com/web/). To locate additional settlements, we used coordinates for healthcare facilities from a list of facilities from the Ghana Health Service. Locations were visually inspected via satellite imagery in Google Earth to confirm the presence of a settlement. We georeferenced each case to the town of residence; if this was unknown then we used the location of the healthcare facility where the patient was evaluated. For our final models, two cases from the urban cycle were georeferenced to healthcare facilities and the remaining were georeferenced to town of residence. To improve precision for the ENMs, we excluded cases that were only referenced at the region or district level.

*Processing YF Occurrence data*

Occurrences were then processed to obtain a final list for model input. We removed duplicate locations and those that lacked epidemiological data. The earliest occurrences used in the model were from 1955. This timeframe was chosen for our models because YF vaccination became required for foreigners in 1945 and a WHO panel of YF experts concluded that cases since 1960 were an important risk factor in Africa [1]. We treated multiple cases at the same location in a given year as a single occurrence. To spatially thin occurrences to account for sampling bias, we used a spatial buffer of 1 km in ArcGIS Pro to remove occurrences that were within 1 km of another occurrence. A final list of 23 locations with laboratory confirmed YF cases with georeferenced coordinates was used as the input data for our models. The locations, coordinates, years, suspected cycles, and references for these coordinates are available in the online dataset. From the 2021 outbreak that started in the West Gonja district we identified 8 locations based on a recent survey of communities with positive YF cases [2]. This study included 22 rural communities with confirmed YF cases. We were able to georeference 12 of these communities, and we used a 1 km buffer to spatially thin these occurrences to 8 locations.

*Covariate data*

We considered covariates that been previously used in models for YF. This included the 19 WorldClim Bioclimatic variables, elevation, human population density, species richness of NHPs (Cercopithecidae), and NDVI. All variables were quantitative variables. Covariates were clipped in ArcGIS Pro using the Admin 0 shapefiles of Ghana from Humanitarian Data Exchange (<https://data.humdata.org/dataset/cod-ab-gha>?). Raster files where then converted to .asc files for modeling. The band statistics tool in ArcGIS pro was used to create a correlation matrix of covariates. Covariates were removed that had |r|>0.7 [3]. We also removed BIO8, 9, 18, and 19 given discontinuities in these layers in sub-Saharan Africa [4]. NHP species richness was considered in initial models but had low contribution to models and caused artifacts given discrete values; therefore, it was removed from the final group of models. The final covariates considered for model comparison included annual mean temperature (BIO1), annual precipitation (BIO12), precipitation in the wettest quarter (BIO16), NDVI, human population density, and elevation.

*Model calibration and evaluation*

Given that we had a small sample size of presence only data, we chose to use Maxent with a leave-one-out cross validation (jackknife) approach [5,6]. We used Maxent as implemented via the maxnet R package, which fits Maxent models using glmnet, and tuned models using the ENMEval package [7]. Our code and data are available with the accompanying R markdown file with the online datasets, available at https://doi.org/10.6084/m9.figshare.24747165.v6 and at https://github.com/sethdjudson/YF_Ghana_Emergence.

Because of our small sample size and goal of exploratory analysis, we chose to simplify our models to avoid overfitting [8]. Therefore, we compared linear, quadratic, and interaction terms as feature classes and regularization multipliers of 1-5. For final model selection among cross-validation results, we first selected models with the lowest 10^th^ percentile omission rate and then used the highest validation AUC value as a tie-breaker [7,9]. The 10th percentile omission rate is the proportion of presence points that fall below the 10^th^ percentile of habitat suitability, and for an ideal model one would expect this to be 0.1 [9]. We also calculated the small sample size version of Akaike Information Criterion (AICc) for further comparison [10]. The selected optimal model using all 23 YF occurrences included three covariates: NDVI, population density, and precipitation in the wettest quarter (BIO16) with quadratic feature classes with a regularization multiplier of 2. The selected optimal model had an average 10^th^ percentile omission rate of 0.13, average cross validation AUC 0.74, and the lowest AICc (delta AICc 0).

We also evaluated another set of models removing the 9 occurrences that were likely due to urban YF. This second set of models contained 14 occurences likely representing where outbreaks began in the savanna cycle. We again evaluated models based on the feature classes and regularization multipliers described above. The selected model from this set of models included annual precipitation (BIO12), precipitation in the wettest quarter (BIO16), NDVI, and human population density with linear feature classes and a regularization multiplier of 3. The selected model had an average 10^th^ percentile omission rate of 0.14, average cross validation AUC 0.82, and delta AICc of 7.5.

The predictions for the selected models containing (A) all YF occurrences and (B) excluding urban occurrences are shown below along with the response curves. The jackknife occurrence locations are also shown. The default cloglog output was used for mapping the predictions in S1 Fig and response curves in S2 Fig.

References

1. Risk assessment on yellow fever virus circulation in endemic countries. [cited 7 Nov 2023]. Available: https://www.who.int/publications-detail-redirect/WHO-HSE-PED-CED-2014-2

2. Inusah A-W, Collins G, Dzomeku P, Head M, Ziblim S-D. Population knowledge, attitudes and practice towards Yellow Fever among nomadic populations: A cross-sectional study in Yellow Fever outbreak Communities in Ghana. [cited 19 Dec 2022]. doi:10.1101/2022.06.14.22276408

3. Feng X, Park DS, Walker C, Peterson AT, Merow C, Papeş M. A checklist for maximizing reproducibility of ecological niche models. Nature Ecology & Evolution 2019 3:10. 2019;3: 1382–1395. doi:10.1038/s41559-019-0972-5

4. Booth TH. Checking bioclimatic variables that combine temperature and precipitation data before their use in species distribution models. Austral Ecology. 2022;47: 1506–1514. doi:10.1111/aec.13234

5. Pearson RG, Raxworthy CJ, Nakamura M, Townsend Peterson a. Predicting species distributions from small numbers of occurrence records: A test case using cryptic geckos in Madagascar. Journal of Biogeography. 2007;34: 102–117. doi:10.1111/j.1365-2699.2006.01594.x

6. Shcheglovitova M, Anderson RP. Estimating optimal complexity for ecological niche models: A jackknife approach for species with small sample sizes. Ecological Modelling. 2013;269: 9–17. doi:10.1016/j.ecolmodel.2013.08.011

7. Kass JM, Muscarella R, Galante PJ, Bohl CL, Pinilla-Buitrago GE, Boria RA, et al. ENMeval 2.0: Redesigned for customizable and reproducible modeling of species’ niches and distributions. Methods in Ecology and Evolution. 2021;12: 1602–1608. doi:10.1111/2041-210X.13628

8. Merow C, Smith MJ, Edwards Jr TC, Guisan A, McMahon SM, Normand S, et al. What do we gain from simplicity versus complexity in species distribution models? Ecography. 2014;37: 1267–1281. doi:10.1111/ecog.00845

9. Radosavljevic A, Anderson RP. Making better Maxent models of species distributions: complexity, overfitting and evaluation. Journal of Biogeography. 2014;41: 629–643. doi:10.1111/jbi.12227

10. Warren DL, Seifert SN. Ecological niche modeling in Maxent: the importance of model complexity and the performance of model selection criteria. Ecological Applications. 2011;21: 335–342. doi:10.1890/10-1171.1
