# Supplementary material for: Yellow fever in Ghana: Predicting emergence and ecology from historical outbreaks
Source: PLOS Glob Public Health. 2024 Oct 21;4(10):e0003337. doi: 10.1371/journal.pgph.0003337 (PMC11493279; doi:10.1371/journal.pgph.0003337)
Supplement: S1 Fig — The predictions based on the default cloglog output for the selected models containing (A) all 23 YF occurrences and (B) 14 occurrences from the savanna cycle are shown. Values closer to 1 indicate a higher relative suitability for YF based on the locations of confirmed human cases and covariates. The georeferenced confirmed YF occurrences are represented by points. (PDF) [file pgph.0003337.s002.pdf]

S1 Fig. Predictions for YF models

A. *Overall YF Model*

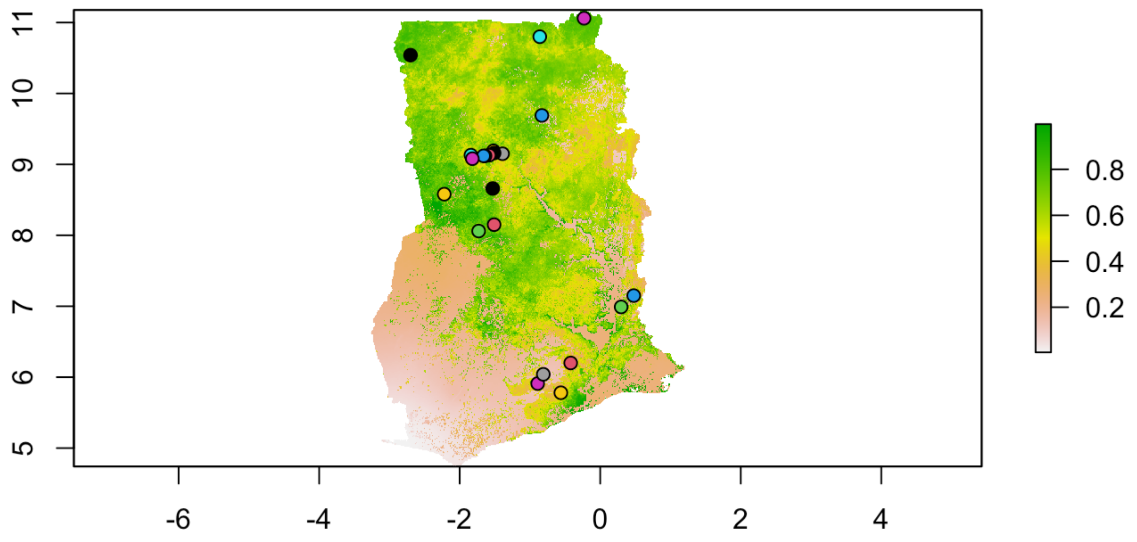

B. *Savanna YF Model*

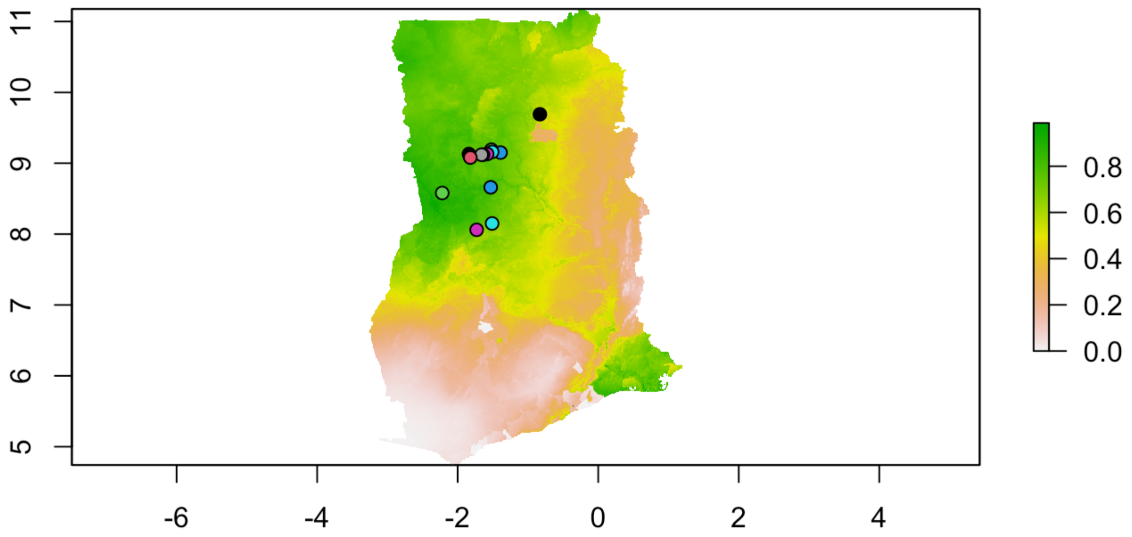

The predictions based on the default cloglog output for the selected models containing (A) all 23 YF occurrences and (B) 14 occurrences from the savanna cycle are shown. Values closer to 1 indicate a higher relative suitability for YF based on the locations of confirmed human cases and covariates. The georeferenced confirmed YF occurrences are represented by points.
