# Supplementary material for: Yellow fever in Ghana: Predicting emergence and ecology from historical outbreaks
Source: PLOS Glob Public Health. 2024 Oct 21;4(10):e0003337. doi: 10.1371/journal.pgph.0003337 (PMC11493279; doi:10.1371/journal.pgph.0003337)
Supplement: S2 Fig — The response curves above visualize the relationship between the probability of YF occurrence and covariates for each model. The shape of the curve indicates how relative suitability changes in response to the covariates. For example, the steeply downward sloping of the response curve for the savanna YF model with population density indicates a greater negative relationship between population density and savanna YF. Note that the standard units for each variable are used except for NDVI (which is rescaled from 0 to 250 by the data source rather than -1 to 1). (PDF) [file pgph.0003337.s003.pdf]

S2 Fig. Response Curves for YF Models

A. *Overall YF model*

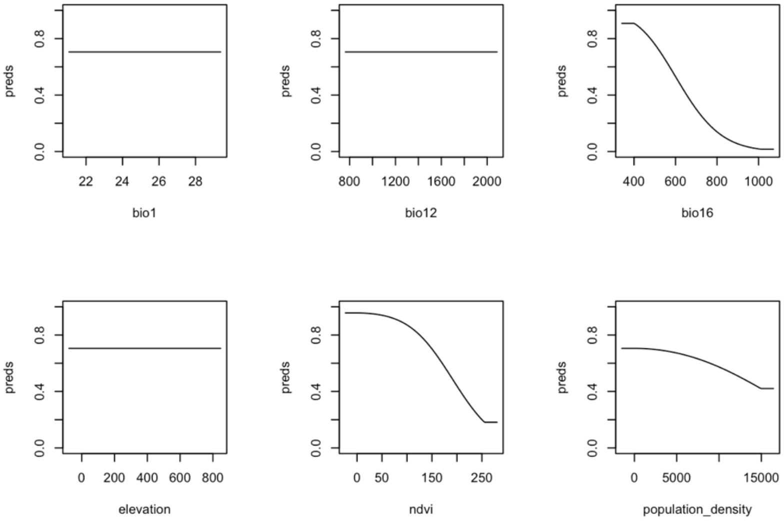

B. *Savanna YF model*

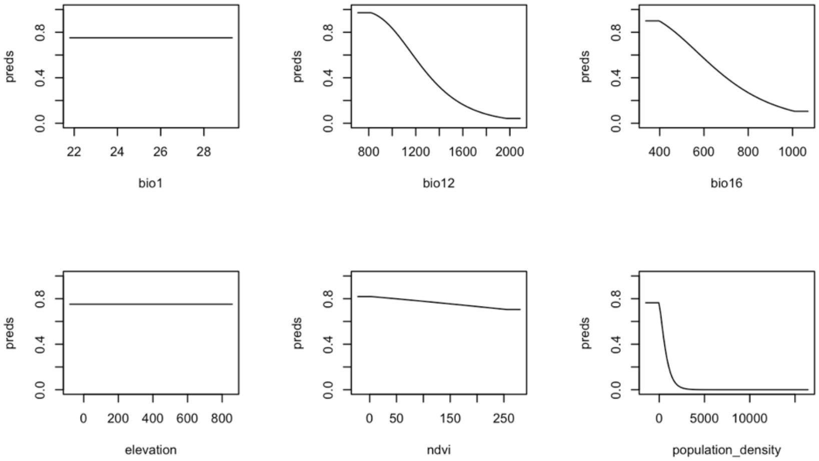

The response curves above visualize the relationship between the probability of YF occurrence and covariates for each model. The shape of the curve indicates how relative suitability changes in response to the covariates. For example, the steeply downward sloping of the response curve for the savanna YF model with population density indicates a greater negative relationship between population density and savanna YF. Note that the standard units for each variable are used except for NDVI (which is rescaled from 0 to 250 by the data source rather than -1 to 1).
